# Supplementary material for: UV photonic integrated circuits for far-field structured illumination autofluorescence microscopy
Source: Nat Commun. 2022 Jul 27;13:4360. doi: 10.1038/s41467-022-31989-8 (PMC9329385; doi:10.1038/s41467-022-31989-8)
Supplement: Supplementary file 1 — Supplementary Information [file 41467_2022_31989_MOESM1_ESM.pdf]

Supplementary information

UV photonic integrated circuits for far-field structured illumination autofluorescence microscopy

Chupao Lin [1,2,6], Juan Santo Domingo Peñaranda[3], Jolien Dendooven[3], Christophe Detavernier[3], David Schaubroeck[4], Nico Boon[5], Roel Baets[1,2] and Nicolas Le Thomas [1,2,7]

[1] Photonics Research Group, INTEC Department, Ghent University-imec, Technologiepark-Zwijnaarde, 9052 Ghent, Belgium

[2]Center for Nano- and Biophotonics, Ghent University, Belgium

[3] Department of Solid State Sciences, CoCooN, Ghent University, Krijgslaan 281/S1, 9000 Ghent, Belgium

[4] Centre of Microsystems Technology (CMST), imec and Ghent University,Technologiepark 126, B-9052 Zwijnaarde, Belgium

[5] Center for Microbial Ecology and Technology (CMET), Ghent University, Coupure Links 653, Building A-9000, Gent, Belgium

[6] Chupao.Lin@UGent.be

[7] Nicolas.LeThomas@UGent.be

Supplementary note 1: Design of the integrated gratings to generate the far-field UV interference fringe patterns

The configuration of structured illumination microscopy (SIM) upgraded with UV photonic integrated circuits (UV-PICs) can be separated into two parts, namely the excitation and collection paths. In this letter, the beams that provide the structured illumination excitation are produced by grating out-couplers integrated on the UV-PICs, the design of which is discussed here. To couple the on-chip guided light to the far-field with the gratings, the phase matching condition needs to be fulfilled between the guided mode and far-field scattered mode: $\vec{\mathbf{k}}_{\mathbf{out}} = \vec{\mathbf{k}}_{\mathbf{in}} \pm p\frac{2\pi}{\Lambda}$, where Λ is the grating pitch, p is an integer, $\vec{\mathbf{k}}_{\mathbf{out}}$ and $\vec{\mathbf{k}}_{\mathbf{in}}$ are the wave vectors of the out-coupled mode and guided mode, respectively. The wave vector $\vec{\mathbf{k}}_{\mathbf{in}}$ is related to the effective refractive index n_{eff} of the guided mode by $\vec{\mathbf{k}}_{\mathbf{in}} = n_{eff}\frac{2\pi}{\lambda_{ex}}\vec{\mathbf{j}}$, with λ_{ex} the excitation wavelength and $\vec{\mathbf{j}}$ a unit vector in the direction of the mode propagation. To achieve a high coupling efficiency in a unique specific scattering angle, the grating out-coupler is designed to couple only the negative 1st scattered order into the free space. It follows that the scattered angle θ is given by $\sin(\theta) = n_{eff} - \frac{\lambda_{ex}}{\Lambda}$ with θ the angle between the $\vec{\mathbf{k}}_{\mathbf{out}}$ and the axis perpendicular to the chip surface. As the numerical aperture of the excitation beam is defined by $\mathrm{NA}_{\mathrm{ex}} = n \cdot \sin(\theta)$, it is directly linked to the grating parameters via $\mathrm{NA}_{\mathrm{ex}} = \frac{\lambda_{ex}}{\Lambda} - n_{eff}$, for the far-field index $n = 1$. The grating out-couplers are fabricated on the 20 µm-wide alumina waveguides adiabatically tapered from 0.7 µm-wide single-mode waveguides. In this way, the guided light keeps propagating in the fundamental transverse electric (TE) mode after the waveguide section of expanding width. In the case of $\lambda_{ex} = 360\ nm$ and alumina film thickness $h = 120\ nm$, the mode effective index n_{eff} of the waveguide gratings is estimated to be $\sim$1.5 using a finite difference eigenmode solver (MODE, Lumerical). As a result, the grating pitches of 180 nm and 150 nm correspond to $\mathrm{NA}_{\mathrm{ex}} = 0.5$ and 0.9, respectively. The etching depth of the gratings is optimized to 30 nm via a two-dimensional finite-difference time-domain method (2D-FDTD, Lumerical) to achieve high coupling efficiency up to 60%, which takes into account the back reflection from the bottom Si/SiO_2 interface.

Supplementary note 2: Experimental structured illumination beam profile

The UV structured illumination generated by the grating out-coupler pairs is imaged with a standard microscope. The focus is located at the plane where the two beams come across above the chip. The focal distance $\mathrm{W_F}$ can be derived from the spacing L between the grating pairs and the numerical aperture $\mathrm{NA}_{\mathrm{ex}}$ of the gratings: $\mathrm{W_F} = \frac{L}{2\tan\left(\mathrm{acrsin}\left(\frac{\mathrm{NA_{ex}}}{n}\right)\right)}$. In the case of $\mathrm{NA}_{\mathrm{ex}} = 0.5$ and $n = 1$, a convenient $\mathrm{W_F}$ can be determined to be 2.4 mm with a spacing L=2.8 mm. The fringe spacing, visibility and field-of-view are critical for the SIM technique, as they determine the maximum theoretical resolution enhancement, the contrast of high frequency features and the imaging throughput respectively. In fig. S1 (a-c), interference fringe patterns with different orientations are generated from the on-chip grating pairs with grating pitch of 180 nm. The measured fringe spacing of 362 nm matches well with the designed numerical aperture $\mathrm{NA}_{\mathrm{ex}} = 0.5$, which validates the robustness of simulation and the well-controlled fabrication technique. Averaging the visibility $\nu = \frac{I_{max} - I_{min}}{I_{max} + I_{min}}$ over the field-of-view (FoV), results in values as high as 0.883, 0.889 and 0.911 for direction D_1, D_2 and D_3 respectively. Their standard deviation values as low as 0.004 are in line with the homogeneity of the beam profile inside the field of view. Such a FoV is estimated

to be 32 µm by 32 µm, as defined by the full-width-at-half-maximum (FWHM) of the quasi-Gaussian profile of the scattered beams. The grating out-coupler is designed to reach both a high irradiance and a large FoV by optimizing the grating modulation etch depth. To further validate the flexibility of the UV-PICs in terms of the direction of the scattered beam, we have designed grating out-couplers with a grating pitch of 150 nm to achieve a numerical aperture $\mathrm{NA_{ex}}$ as high as 0.9. Due to the imperfection in the MMI and grating fabrication, the imbalanced intensity coupled out from two gratings for each pair leads to relatively lower but high enough visibilities for the different orientations D_1, D_2 and D_3, with values of 0.730 ± 0.005 , 0.773 ± 0.008 and 0.737 ± 0.003 respectively (see fig S1(e-f)). The field of view of 30 µm by 30 µm is similar with that of the scattered beam with numerical aperture $\mathrm{NA_{ex}} = 0.5$ and the lower fringe spacing of 199 nm agrees with numerical aperture $\mathrm{NA_{ex}} = 0.9$.

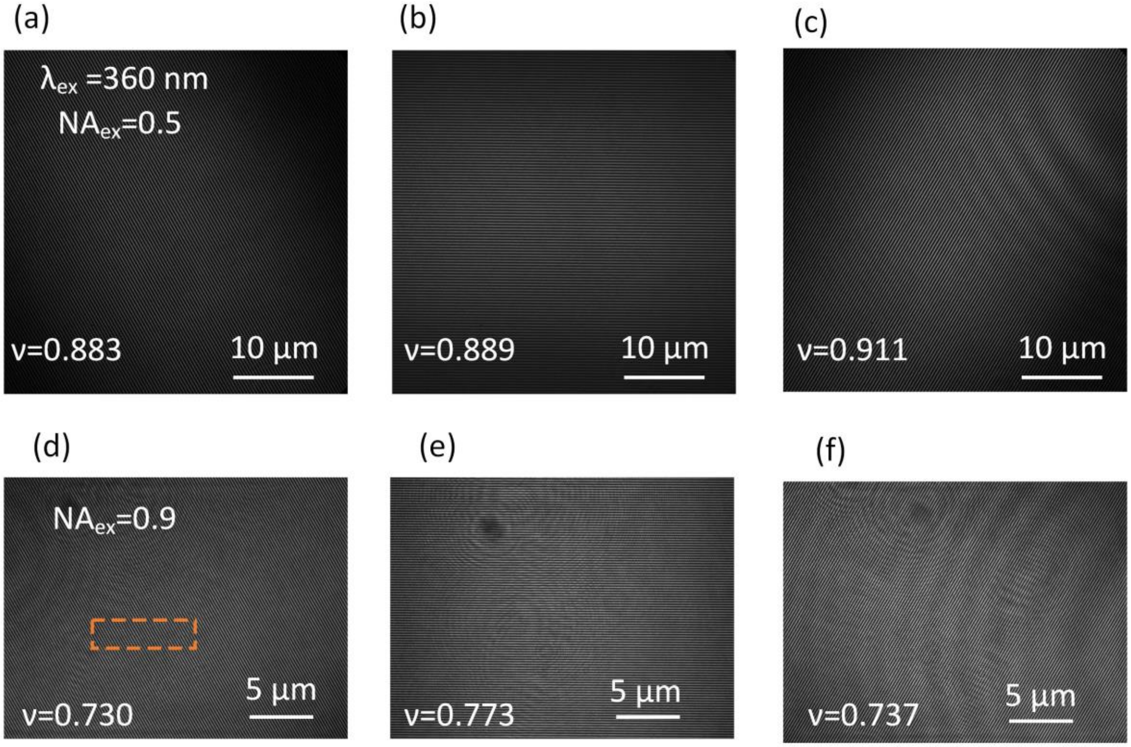

Figure S1 Optical images of the interference fringe pattern at the object plane produced by two UV beams coupled out from the on-chip grating pairs along different orientations, namely D_1, D_2 and D_3 respectively (see main text). (a-c) A grating pitch of 180 nm corresponding to NA_{ex} = 0.5 results in a fringe spacing of 362 nm and a field of view (FoV) = 32 µm x 32 µm. (d-f) A grating pitch of 150 nm corresponding to NA_{ex} = 0.9 results in a fringe spacing of 199 nm and FoV = 30 µm x 30 µm.

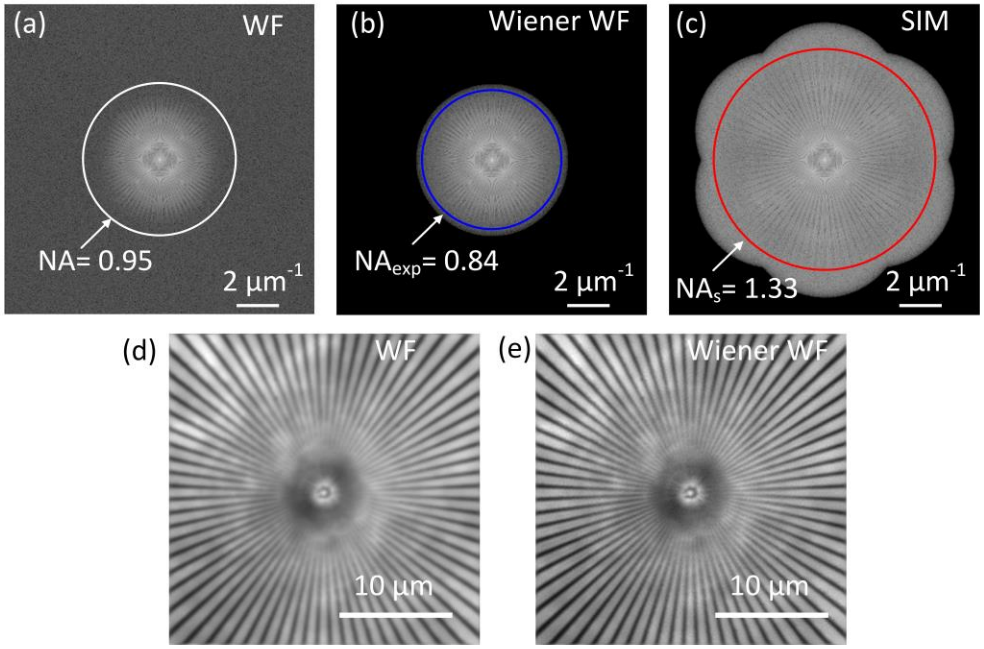

Figure S2 Fourier domain images of the sector star target with fluorescent dyes obtained through fast Fourier transform (FFT) from real space domain images. (a) The real space image is acquired by the wide-field (WF) microscopy and subjected to experimental noises. The white circle indicates the theoretical bandwidth of the microscope objective with NA= 0.95. (b) WF image with implementation of a Wiener filter to minimize the impact of the experimental noise. The white circle in (a) locates the ideal spatial frequency bandwidth of the NA= 0.95 microscope objective. The blue circle in (b) indicates the actual bandwidth achieved with the NA= 0.95 microscope objective in the experiment, which corresponds to that of a microscope objective of NA= 0.84 due to the presence of noise. (c) Reconstructed super-resolved SIM image, see space domain in figure 3(b) and 3(d) of the main text. Resolution enhancement can be clearly seen from the extended signal region compared with (b). The maximum resolved spatial frequency is highlighted by the red cycle, corresponding to the bandwidth of an objective with synthetic numerical aperture NA_S=1.33. (d-e) WF real space images corresponding to the FFT of (d) and (e), respectively.

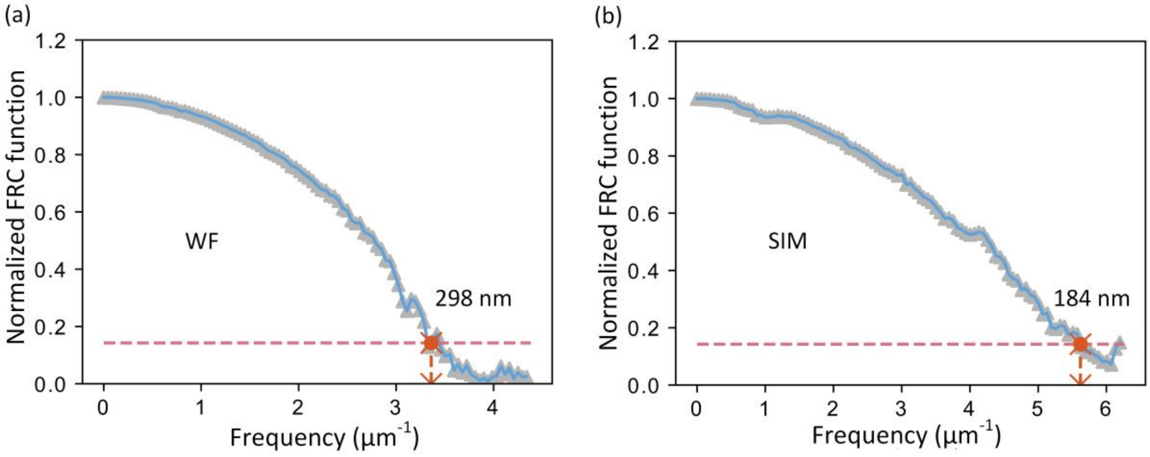

Figure S3. Fourier ring correlation curves. (a) WF in fig. 3(a), (b) SIM without deconvolution

Supplementary note 3: Analysis of the image reconstruction process in the Fourier domain

The high quality of the structured illumination generated by the UV-PICs is expected to achieve 'artifact-free' super-resolved image reconstruction in SIM microscopy. Any inaccuracy in the estimation of the parameters, such as the spatial frequency of the excitation beams, the relative phase of the beams and the visibility of structured illumination for each frame contributes however to artifacts in the reconstructed image. Among these factors the relative phase error contributes to the incomplete disentanglement between low and high frequency bands and then leads to periodical artifacts stemming from the structured illumination. It is convenient to inspect the artifacts in the Fourier domain by monitoring the corresponding residue peaks in the disentangled frequency bands. In practice, the acquired images are generally corrupted by the experimental noise and the signal-to-noise ratio (SNR) of each acquired frame determines the experimental resolution limit in microscopy. As shown in fig. S2(a), the strong bandwidth-limited signal of the WF image in the Fourier domain can be easily seen in the center part and the noise level can be estimated at the outer part. To minimize the impact of the noise and properly estimate the spatial resolution limit for the conventional WF microscopy, the WF image is filtered through a conventional Wiener filter. The achieved resolution limit is indicated by the blue circle of radius 3.27 μm^{-1} in fig. S2(b), corresponding to a minimum resolvable spacing of 192 nm in the real space domain. Figure S2(c) shows the reconstructed Fourier domain of the super-resolved SIM image. The features of the sector star target are clearly extended in the expanded bandwidth of the UV-PIC based SIM microscopy. It is worth noting that there are no visible residue peaks located at the spatial frequency of structured illumination, which reveals the high accuracy in parameter estimation, the high quality of the structured illumination and the artifact-free reconstruction of the image. The raw and filtered WF images are compared in fig. S2(d) and (e), respectively. The Wiener filter removes the noise outside the bandwidth of the OTF and enhances the contrast of the high-frequency signal but has no impact on the optical resolution limit here. The Fourier ring correlation (FRC) method [1] that is a standardized approach in the field of microscopy is implemented to confirm the resolution limit of WF and SIM images. The FRC curves of WF and SIM images without applying deconvolution are plotted in Fig. S3. The optical resolutions that are extracted by the intersections between the FRC curves with a conventional threshold of 1/7 are 298 nm and 184 nm for WF and SIM, respectively. The results are in line with the resolutions determined by the FFT peak analysis in Fig. 3(f), namely resolutions of 305 nm and 192 nm, respectively, where a 3σ criterium has be used to define the threshold.

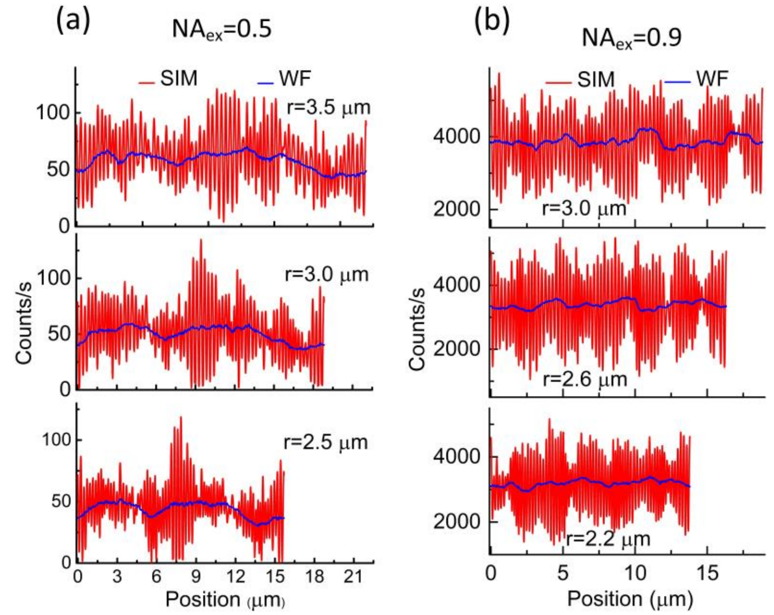

Figure S4 Intensity profiles along different circular cross-sections of the optical images of the sector star target acquired by WF (blue) and SIM (red) microscopy with an NA_{co}=0.95 objective. (a) NA_{ex}=0.5. (b) NA_{ex}=0.9.

Supplementary note 4: Extended analysis of the high spatial frequency features resolved by UV-PICs based SIM microscopy

The intensity profiles in the fig. 3(e) and fig. 4(g) of the main text show the maximum resolvable spatial frequency in the WF and SIM microscopy, for the case of NA_{ex}= 0.5 and 0.9 respectively. To further validate the UV-PIC based SIM technique, more cross-section profiles along different radii r are plotted in fig. S4(a) and (b) for NA_{ex}= 0.5 and 0.9, respectively. The visibility of the oscillation at $r = 3.5\ \mu m$ in fig. S4(a) is weak with the WF microscopy but still distinguishable, which can be confirmed by the presence of the corresponding peak in the Fourier domain (see fig. 3(f) of the main text). This result implies a resolution limit of 305 nm for the conventional WF microscopy. In the case of smaller circular cross-sections of radii $3.0\ \mu\mathrm{m}$ and $2.5\ \mu\mathrm{m}$, which correspond to grating pitches of 262 nm and 218 nm, respectively, the SIM microscopy easily resolves the grating features while WF microscopy cannot. In the case of NA_{ex}= 0.9 and NA_{co}= 0.95, the UV-PIC based SIM technique experimentally reaches a resolution limit as low as 166 nm, see fig. 4(g) of the main text. More intensity profiles at $r = 3.0\ \mu\mathrm{m}$, $2.6\ \mu\mathrm{m}$ and $2.2\ \mu\mathrm{m}$ are plotted here in fig. S4(b), demonstrating that the oscillations with different frequencies are observable in the SIM images but indistinguishable in WF images and that the resolution enhancement is achieved with the UV-PIC based SIM technique.

In order to check the reliability of the reconstruction outcome, the Fourier transforms of the intensity profiles of the WF and SIM images are compared with those of the SEM images. As shown in fig. S5, the position of the peak maximum of the SIM curve (red) matches well with that of SEM (black), validating that the SIM approach retrieves properly the true profile of the object. In contrast, no peak can be

observed in the WF images (blue) since the grating features along these circular cross-sections oscillate at frequencies beyond the maximum bandwidth in the WF microscopy.

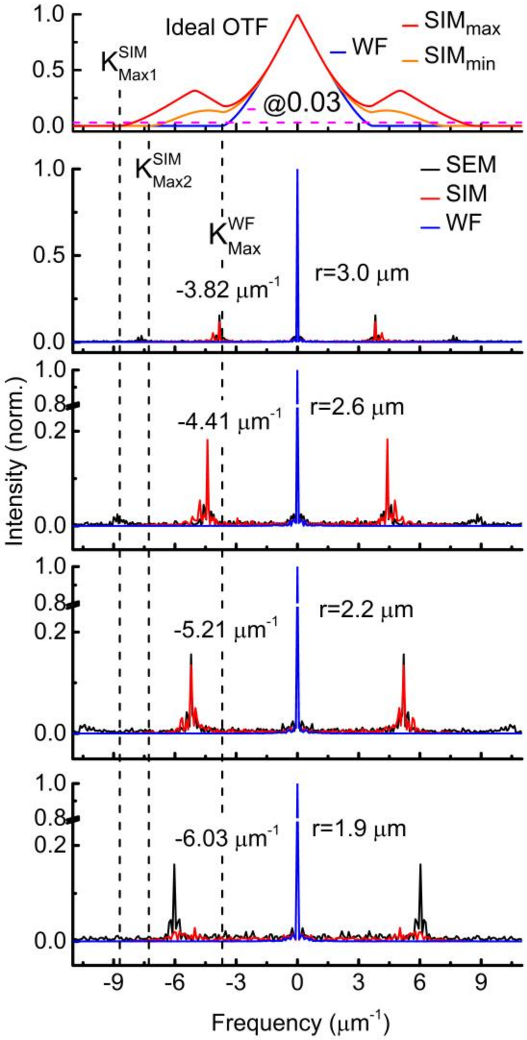

Fig. S5 Fourier transforms of the line profile in fig. S4(b) and fig. 4(g) of the main text. The profiles of the ideal optical transfer function (OTF) in the WF and UV-PICs based SIM microscopy are plotted at the top, where K_{Max}^{WF}, K_{Max1}^{SIM} and K_{Max2}^{SIM} are defined by the maximum bandwidths in the WF microscopy, in the SIM microscopy along the illumination orientation and along the direction perpendicular to illumination orientation respectively. The dashed purple line sets the intensity threshold over which the signal is detectable.

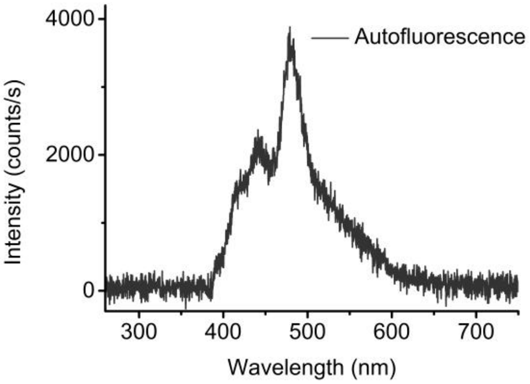

Fig. S6 Fluorescence spectrum of yeast cells with UV excitation at λ= 360 nm.

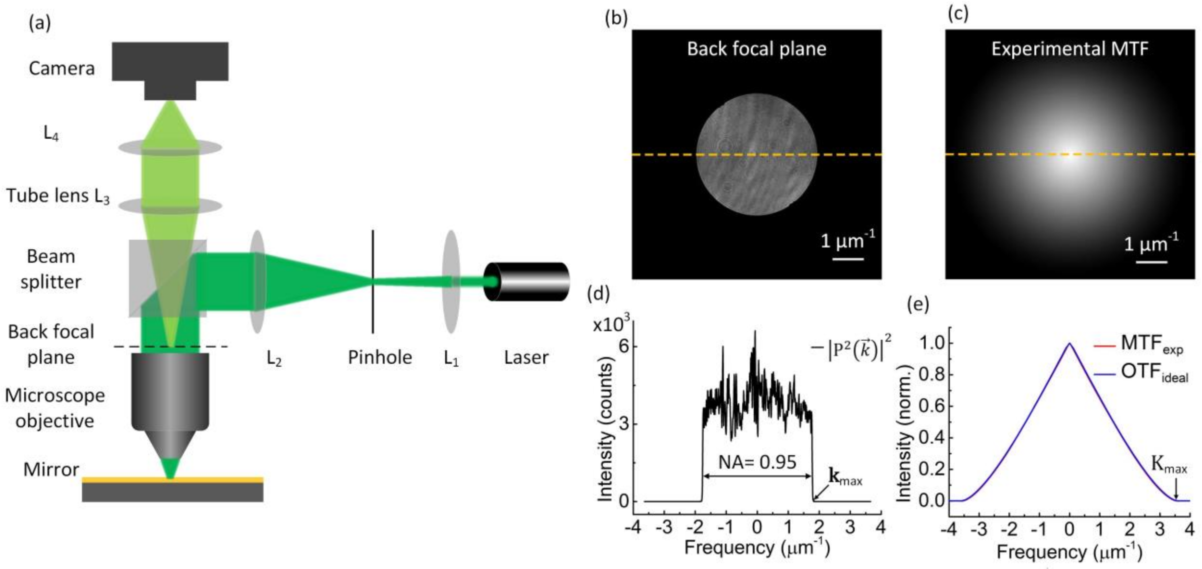

Fig. S7 Modulation transfer function (MTF) measurement. (a) Schematic of the experimental setup used to determine the MTF. The numerical aperture of the microscope objective is $NA_{col} = 0.95$ and the working wavelength $\lambda = 532\ nm$. (b) Intensity profile of the squared pupil function measured in back-focal plane, namely $\left|\mathrm{P}^2(\vec{\mathbf{k}})\right|^2$, with $\mathrm{P}(\vec{\mathbf{k}})$ the complex pupil function and $\vec{\mathbf{k}}$ the wave vector in the plane perpendicular to the optical axis. (c) Retrieved MTF of the microscope objective. (d) Profile in linear scale along the dashed orange line in (b) with the bandwidth given by $\mathrm{k}_{max} = \frac{2\pi}{\lambda} NA_{col}$. (d) Normalized intensity profiles of the theoretical optical transfer function (blue) and the measured MTF (red), with the maximum of the bandwidth given by $\mathrm{K}_{max} = \frac{2\pi}{\lambda} \times 2NA_{col}$.

Supplementary note 5: Experimental MTF measurement

To reconstruct a super-resolved SIM image, the prior knowledge of the optical transfer function (OTF) is needed. The modulus and phase of the OTF are named modulation transfer function (MTF) and phase transfer function (PTF), respectively. As we have shown that the latter is constant (see main text), the OTF

can be directly retrieved from the knowledge of the MTF. To experimentally determine the MTF, we have built a setup that acquires the intensity of the squared pupil function $\mathrm{P}^2(\vec{\mathbf{k}})$, with $\vec{\mathbf{k}}$ the wave vector in the plane perpendicular to the optical axis. This measurement setup is similar to the one used in ref. [2] except that a mirror replaces the opposite microscope objective and an extra lens images the back focal plane of the microscope objective, as illustrated in fig. S7(a). Besides, the lens L_1 focuses the emission from a green laser on a 10 µm-diameter pinhole in order to generate a Gaussian beam. The laser operating at a wavelength of 532 nm close to the emission wavelength of the fluorophores described in the main text. The second lens L_2 collimates the central spot of the field diffracted by the pinhole to provide a homogenous illumination pattern. This beam is reflected by a 50:50 beam splitter and focused on the mirror by the microscope objective. The Fourier transform $\widetilde{\mathrm{E}}_{in}(\vec{\mathbf{k}})$ (up to a phase term) of the field located at the pinhole is retrieved in the back-focal plane of L_2, which is also the back-focal plane of the microscope objective. As the beam reflected by the mirror passes through the objective twice, the output field in the back focal plane is given by $\widetilde{\mathrm{E}}_{out}(\vec{\mathbf{k}}) = \mathrm{P}(\vec{\mathbf{k}}) \cdot \mathrm{P}(\vec{\mathbf{k}}) \cdot \widetilde{\mathrm{E}}_{in}(\vec{\mathbf{k}})$. As the input beam is homogenous and much larger than the aperture size of the objective, it is considered as constant inside the pupil, and consequently $\widetilde{\mathrm{E}}_{out}(\vec{\mathbf{k}}) = \mathrm{P}^2(\vec{\mathbf{k}})$. In the imaging path, a telescope made of two lenses, namely the tube lens L_3 and the lens L_4, images the back-focal plane of the microscope objective. As cameras provide the intensity of the field, the acquired image corresponds to the intensity of squared pupil function, namely $\left|\mathrm{P}^2(\vec{\mathbf{k}})\right|^2$ (see fig. S7(b)). Excluding small intensity fluctuations resulting from residual imperfections of the optical components of the set-up, the experimental intensity profile in the back-focal plane is in line with the ideal pupil function (see fig. S7(d)). Considering that the phase of the experimental OTF is constant (see main text), the phase of the pupil function $\mathrm{P}(\vec{\mathbf{k}})$ can also be taken as constant. As a result, the normalized $\mathrm{MTF} = \frac{|\mathrm{P}(\vec{\mathbf{k}}) \otimes \mathrm{P}^*(\vec{\mathbf{k}})|}{\int |\mathrm{P}(\vec{\mathbf{k}})|^2 d\vec{\mathbf{k}}}$ can be expressed as $\mathrm{MTF} = \frac{|\mathrm{P}(\vec{\mathbf{k}})| \otimes |\mathrm{P}(\vec{\mathbf{k}})|}{\int |\mathrm{P}(\vec{\mathbf{k}})|^2 d\vec{\mathbf{k}}}$, where $\otimes$ represents for convolution operator and $\mathrm{P}^*(\vec{\mathbf{k}})$ is the complex conjugate of $\mathrm{P}(\vec{\mathbf{k}})$. From the modulus of the pupil function, we can then retrieve the MTF as shown in fig. S7(c), which matches well with the ideal one and justifies the use of the ideal OTF in the SIM reconstruction process.

[1] S. Koho, G. Tortarolo, M. Castello, T. Deguchi, A. Diaspro, and G. Vicidomini, "Fourier ring correlation simplifies image restoration in fluorescence microscopy," *Nat. Commun.,* **10**,3103 2019.

[2] J. H. Joachim Wesner, and Thomas Sure, "Reconstructing the pupil function of microscope objectives from the intensity PSF," *Proc. SPIE, Current Developments in Lens Design and Optical Engineering III*, 4767, 2002.
