# Supplementary material for: UV photonic integrated circuits for far-field structured illumination autofluorescence microscopy
Source: Nat Commun. 2022 Jul 27;13:4360. doi: 10.1038/s41467-022-31989-8 (PMC9329385; doi:10.1038/s41467-022-31989-8)
Supplement: Supplementary file 3 — Description of Additional Supplementary Files [file 41467_2022_31989_MOESM3_ESM.pdf]

Supplementary Dataset 1: In this supplementary dataset, we provide the reconstruction algorithm that has been used to reconstruct the super-resolved SIM images from nine raw frames. The source data (.tif) of figures 3 and S2 are provided as an example to test the reconstruction code.

readme.txt : Introduction of the operation of the reconstruction code

Main.m: The main code to execute the reconstruction.

LowK_disentangle.m: Algorithm to disentangle the frequency bands.

LowK_Freq.m: Algorithm to estimate the spatial frequency of the structured illumination.

LowK_phase.m: Algorithm to estimate the phase of the structured illumination.

PatternPhaseOpt.m : Compute the cross-correlation between a 2D sinusoidal function and raw images.

PhaseKai2opt.m: Compute autocorrelation of FT of raw SIM images.
