# Supplementary figures and images for: UV photonic integrated circuits for far-field structured illumination autofluorescence microscopy
Source: Nat Commun. 2022 Jul 27;13:4360. doi: 10.1038/s41467-022-31989-8 (PMC9329385; doi:10.1038/s41467-022-31989-8)

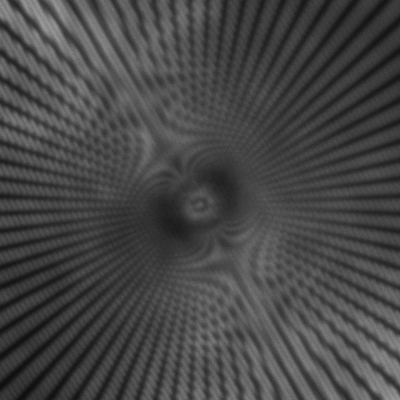

Supplement: Supplementary file 4 — Supplementary Dataset 1 [file 41467_2022_31989_MOESM4_ESM.zip › dapa.tif]

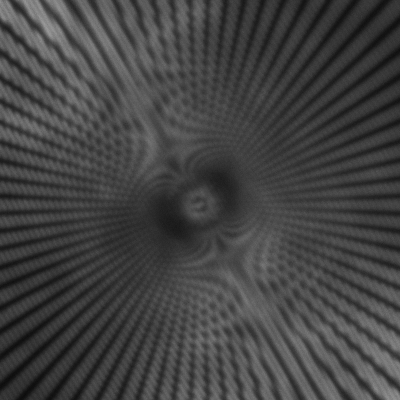

Supplement: Supplementary file 4 — Supplementary Dataset 1 [file 41467_2022_31989_MOESM4_ESM.zip › dapb.tif]

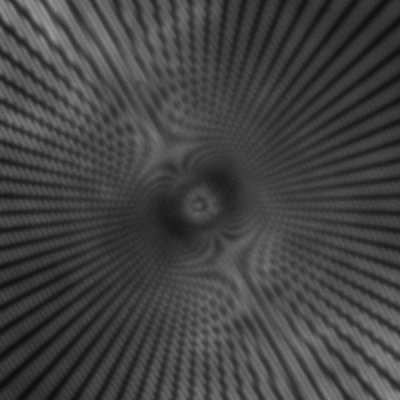

Supplement: Supplementary file 4 — Supplementary Dataset 1 [file 41467_2022_31989_MOESM4_ESM.zip › dapc.tif]

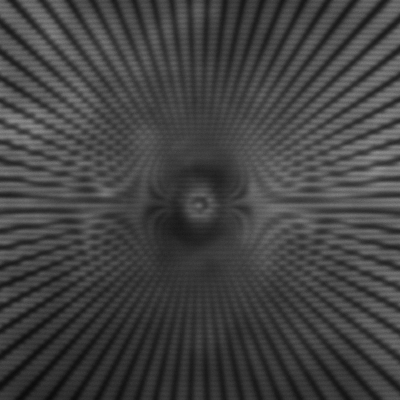

Supplement: Supplementary file 4 — Supplementary Dataset 1 [file 41467_2022_31989_MOESM4_ESM.zip › dbpa.tif]

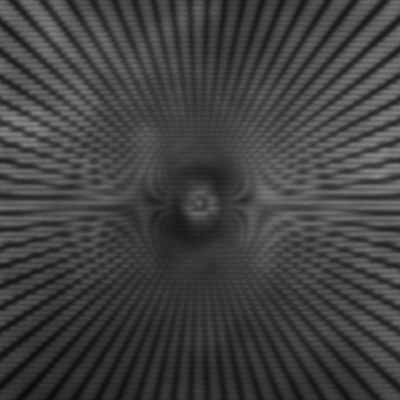

Supplement: Supplementary file 4 — Supplementary Dataset 1 [file 41467_2022_31989_MOESM4_ESM.zip › dbpb.tif]

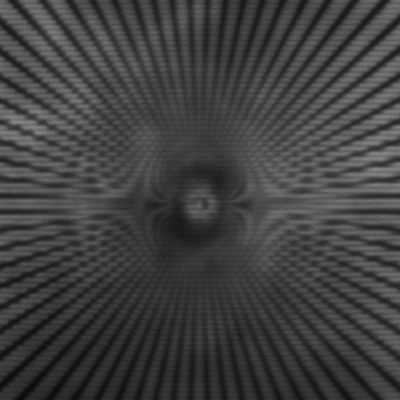

Supplement: Supplementary file 4 — Supplementary Dataset 1 [file 41467_2022_31989_MOESM4_ESM.zip › dbpc.tif]

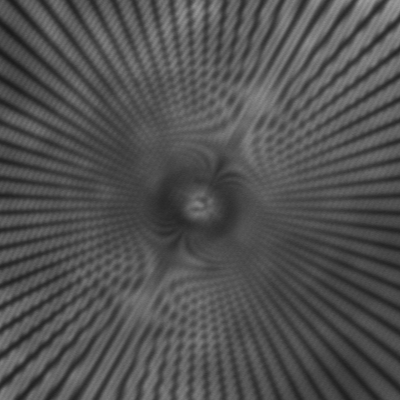

Supplement: Supplementary file 4 — Supplementary Dataset 1 [file 41467_2022_31989_MOESM4_ESM.zip › dcpa.tif]

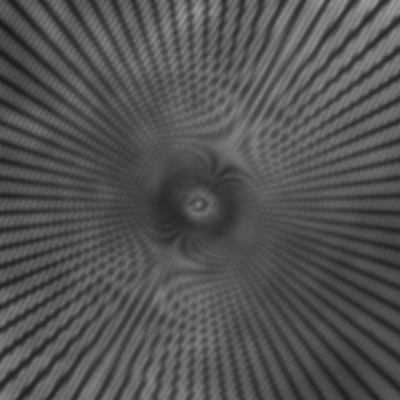

Supplement: Supplementary file 4 — Supplementary Dataset 1 [file 41467_2022_31989_MOESM4_ESM.zip › dcpb.tif]

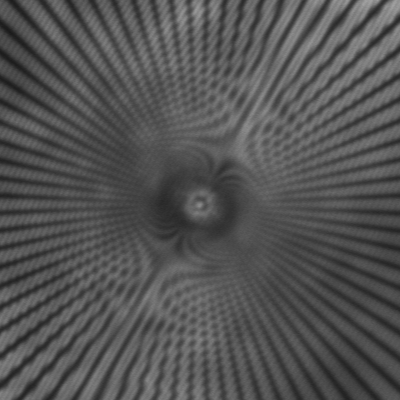

Supplement: Supplementary file 4 — Supplementary Dataset 1 [file 41467_2022_31989_MOESM4_ESM.zip › dcpc.tif]
